# Blood cadmium and volume of uterine fibroids in premenopausal women

**DOI:** 10.1186/s40557-017-0178-8

**Published:** 2017-06-22

**Authors:** Shinhee Ye, Hye Won Chung, Kyungah Jeong, Yeon-Ah Sung, Hyejin Lee, So Yun Park, Hyunjoo Kim, Eun-Hee Ha

**Affiliations:** 1 Department of Occupational and Environmental Medicine, School of Medicine, Ewha Womans University, Seoul, South Korea; 2 Department of Obstetrics and Gynecology, School of Medicine, Ewha Womans University, Seoul, South Korea; 3 Department of Internal Medicine, School of Medicine, Ewha Womans University, Seoul, South Korea; 4 Department of Occupational and Environmental Medicine, Ewha Womans University Mokdong Hospital, Seoul, South Korea

**Keywords:** Heavy metals, Uterine fibroids, Premenopausal women

## Abstract

**Background:**

A number of studies have found associations between heavy metals and uterine fibroids, but the results are inconsistent. Here, we conducted this research to demonstrate the relationships between blood heavy metal concentrations and uterine fibroid volume as well as the rate of uterine fibroid presence.

**Methods:**

In a cross-sectional study, we collected data from 308 premenopausal women aged 30–49 years in Seoul; uterine fibroids are ascertained by past history of myomectomy and pelvic ultrasonography. In the analytic phase, we first analyzed the presence of the fibroids and the concentrations of heavy metals via logistic regression. In subgroup analysis, we used simple and multiple linear regression analyses to examine the associations between heavy metals and uterine fibroid volume.

**Results:**

There was no connection between the heavy metal concentrations and the presence of uterine fibroids, but the odds of women having fibroids were higher with three particular metals. In subgroup analysis, the association between blood cadmium concentrations and uterine fibroid volume was statistically significant (adjusted beta coefficient = 2.22, 95% confidential interval: 0.06–4.37). In contrast, blood mercury and lead concentrations were not significantly associated with uterine fibroid volume.

**Conclusions:**

Our findings are the first that we know to report the association of blood cadmium concentrations with the volume of uterine fibroids. We expect that our findings will be used as evidence for supporting policies to improve premenopausal Korean women’s health.

## Background

Uterine fibroids cause serious symptoms such as uterine bleeding and pelvic pain [1], resulting in major surgery (e.g., hysterectomy). Nearly 70% of Caucasian women show the cumulative incidence of uterine fibroids by the age of 50 [2], and the rate also applies to Asian women [3].

Although the precise causes of fibroids are still unknown, they have been assumed to be associated with reproductive history, body mass index (BMI), and smoking [4]. Fibroids are known for estrogen-dependent diseases, where diseased tissues show more estrogen receptor-α (ERα) than non-diseased tissues [5–7]. Because fibroids are hormonally related, it has been hypothesized that exposure to exogenous estrogens is related to their development and size increase [8, 9].

Metalloestrogens are metals that are known to activate the estrogen receptor in the absence of estradiol [10]. In both in vitro and in vivo studies, metalloestrogens have been identified as the cause of ERα activation [11–13]. Among metalloestrogens, the following three heavy metals have been reported as toxic to people: cadmium, lead, and mercury. The main sources of exposure to each metal are as follows: cadmium exposure is primarily through cigarette smoke, air pollution, and contaminated food [14]; lead exposure is through lead-based paint, contaminated soil, and dust [15]; and mercury exposure is through fish consumption, air pollution, and dental amalgams [16].

Two epidemiologic studies have found the associations between blood heavy metal concentrations and the existence of uterine fibroids, but the results are not consistent [17, 18]. In addition, Korean women are vulnerable to conventional heavy metal exposure because they consume so much rice and seafood [19, 20]. Hence, here we epidemiologically conduct research to demonstrate the relationships between heavy metal concentration and uterine fibroid volume, mainly based on cell proliferation, as well as the rate of uterine fibroids.

## Methods

### Study participants

Using a cross-sectional study, we conducted research on the effects of exposure to environmental risk factors in premenopausal women in Seoul. The data were collected for three months (September to November 2014) to clarify how the environment exposure affects their health.

### Recruitment and informed consent

The women volunteered following a notice on the bulletin board at the Ewha Womans University Medical Center, a support center for healthy families, a community health center, a community service center, and a community blog for mothers. The study protocol was approved by the institutional review board at Ewha Womans University Medical Center, and written informed consent was obtained from all of the women.

### Data and bio-specimen collection

We collected data from 308 premenopausal women in the Republic of Korea, the age range of 30 to 49. For the data analysis, we did not include pregnant or breastfeeding women, whose heavy metal levels at the time might have been influenced by these circumstances and might have been less representative of heavy metal levels at the time of diagnosis [21, 22]; women who had received hysterectomies were also excluded from the analysis. Ultimately, we conducted the statistical analysis with 288 women, 46 with fibroids and 242 without.

In order to clarify the statistical significance between uterine fibroid volume and heavy metals, we also performed subgroup analysis among women with fibroids. Because there was bias resulting from the myomectomy histories of women who did not have a medical record of their uterine fibroid volumes, we conducted our subgroup analyses only with women who had not received a myomectomy (*n* = 40).

We collected the data from interview questionnaires, physical examinations, pelvic ultrasonography, and blood samples; we collected basic health information such as socio-demographic characteristics, prior medical history, reproductive health status, psychosocial status, health behavior, height, weight, and environmental exposure with the assistance of trained researchers using questionnaires.

We collected the data on the presence and volume of the women’s uterine fibroids through pelvic ultrasonography. Specifically, pelvic ultrasounds were performed using the ProSound Alpha 7 ultrasound system (Hitachi Aloka Medical, Tokyo, Japan). We also sampled blood in order to measure the levels of cadmium, mercury and lead; we collected the whole-blood samples in EDTA-treated tubes and stored the mat 4–8 °C throughout the entire analysis period. We conducted these analyses within three days of storage at the Seoul Medical Science Institute.

### Analysis of heavy metals in whole blood

We analyzed each heavy metal as follows: We analyzed cadmium levels using graphite furnace atomic absorption spectrophotometry. The limit of quantification (LOQ) was 0.100 μg/L, and no sample had a cadmium level below the LOQ; we analyzed mercury by inductively coupled plasma mass spectrometry. The LOQ was 0.008 μg/L, and no sample had a mercury level below that; we analyzed lead levels by graphite furnace atomic absorption spectrophotometry. The LOQ was 0.030 μg/dL, and again, no sample had a lead level below the LOQ. Standardized quality-control procedures were carried out for the laboratory analyses, including internal and external controls. For each series of analyses, internal quality control was used. Centers for Disease Control and Prevention (CDC)’s Lead and Multi-element Proficiency (LAMP) external quality program were periodically conducted for the precision and accuracy of cadmium, lead, and mercury level measurement. Additional external quality control for lead measurement was executed by College of American Pathologists (CAP) program.

### Uterine fibroid ascertainment

Diagnosis of uterine fibroids was based on pelvic ultrasonography and two questions: “Have you ever undergone an operation in the past?” and “If you have ever undergone an operation, what was the operation?”; the pelvic sonograms were taken by a well-trained gynecologist. If a participant had undergone a myomectomy in the past or was diagnosed with uterine fibroids by pelvic ultrasonography during our research, we categorized that woman as having uterine fibroids. In addition, if we could identify fibroids during our research based on the pelvic sonograms, we also gathered data on the fibroids volumes. When study participants had multiple uterine fibroids, we selected the largest fibroids by volume as the dominant or marker fibroids for demonstrating the association between blood heavy metal concentrations and uterine fibroid volumes. We calculated fibroid volume by measuring the longest and shortest diameters to determine an ellipsoid shape [23].

### Statistical analysis

All statistical analyses were performed in SAS 9.3 version. Descriptive analyses included evaluating data completeness and comparing women with and without fibroids in relation to socio-demographic, medical, and reproductive history characteristics. We used Chi-square tests to determine statistical significance for the categorical variables. After, the geometric means and standard deviations for the three heavy metal concentrations were calculated by general characteristics and disease status of study participants. Kruskal-Wallis tests were also used to determine statistical significance among non-normally distributed continuous variables. Before moving onto inferential analysis, all concentrations of blood heavy metal and the volume of the largest fibroids were log transformed due to non-normal distribution; this transformation normalized the distributions. In the analytic phase, we first analyzed the presence of uterine fibroids and the heavy metal concentrations via logistic regression. Second, we used simple and multiple linear regressions for the subgroup analyses of the associations between heavy metal concentrations and uterine fibroid volumes. Third, the association between quartiles of heavy metals concentrations and uterine fibroid volumes was assessed using simple and multiple linear regression. The possible confounders were age as continuous, BMI as continuous, the age at menarche as continuous, gravidity categorized as 0, 1, 2, and more than 3, birth control pill administration history dichotomized as never versus used, regularity of menstrual cycle dichotomized as regular versus irregular, hemoglobin level dichotomized as anemia versus non-anemia (<12 g/dL versus ≥12 g/dL), and serum cotinine dichotomized as no versus passive or active exposure (<10 ng/mL versus ≥10 ng/mL).

## Results

**Fig. 1:**
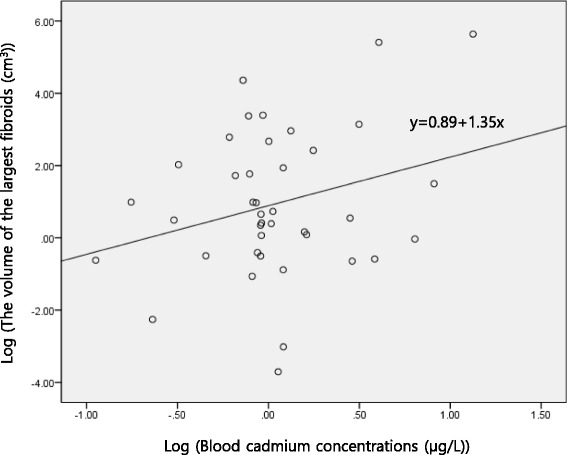
Correlation graph between blood cadmium concentrations and the volume of the largest fibroids

In our study, the prevalence of uterine fibroids was 16.0%. Table 1 shows that the women with uterine fibroids were older than the women without fibroids. Table 2 shows that the research women’s mean blood concentrations of cadmium, lead, and mercury were 0.98 μg/L, 1.36 μg/dL, and 1.61 μg/L, respectively. Women in their 40s had significantly higher cadmium and mercury concentrations than those in their 30s (Table 2). Obese women had a significantly higher mercury concentration than non-obese women and women with anemia had a significantly lower cadmium concentration than women without anemia (Table 2). Although the women with uterine fibroids showed higher blood concentrations of the heavy metals than the women without uterine fibroids, there was no significance between the concentrations and the presence of fibroids, also shown in Table 2. Table 3 shows that there was no significance between the concentrations and the presence of fibroids either before or after adjustment, but the odds of having these three heavy metals in their blood were higher among women who did have uterine fibroids. Tables 4 and 5 shows the results of the subgroup analysis of the women who were found to have uterine fibroids via pelvic ultrasonography. In that subgroup, the mean volume of the largest fibroids was 19.6 ± 56.1 cm^3^ (range: 0.02–280.55cm^3^). In Table 4, the association between blood cadmium concentrations and the volume of the largest fibroids was statistically significant after adjusting for confounders (*P* < 0.05) and the correlation graph between blood cadmium concentrations and the volume of the largest fibroids was represented in Fig. 1. In contrast, blood mercury and lead concentrations were not significantly associated with the volume of the largest fibroids either before or after adjusting for confounders (Table 4). In Table 5, although dose–response association between blood cadmium concentrations and the volume of the largest fibroids did not show a statistically significant, the upper quartile of blood cadmium concentrations had the highest β coefficient.

**Table 1:** General characteristics of study population

Variables	All subjects (*N* = 288)	Women with uterine fibroids (*N* = 46)	Women without uterine fibroids (*N* = 242)	*P*
	Number (%)	Number (%)	Number (%)	
Age				
<40 years old	218 (75.7)	25 (54.4)	193 (79.8)	<0.001
≥40 years old	70 (24.3)	21 (45.7)	49 (20.3)	
Education				
≤High school	92 (31.9)	18 (39.1)	74 (30.6)	0.333
≥University	196 (68.1)	28 (60.9)	168 (69.4)	
Income/monthly				
<4,000,000	156 (54.4)	26 (56.5)	130 (53.9)	0.873
≥4,000,000	131 (45.6)	20 (43.5)	111 (46.1)	
Marital status				
Married/living with partner	282 (98.3)	44 (95.7)	238 (98.8)	0.390
Not married/divorced	5 (1.7)	2 (4.4)	3 (1.2)	
Body mass index				
Non-obesity (<25 kg/m^2^)	245 (85.1)	38 (82.6)	207 (85.5)	0.776
Obesity (≥25 kg/m^2^)	43 (14.9)	8 (17.4)	35 (14.5)	
Hemoglobin				
Anemia (<12 g/dL)	263 (91.3)	39 (84.8)	224 (92.6)	0.152
Non-anemia (≥12 g/dL)	25 (8.7)	7 (15.2)	18 (7.4)	
Serum cotinine				
No exposure (<10 ng/ml)	268 (93.1)	41 (89.1)	227 (93.8)	0.409
Passive/Active/Heavy smoking (≥10 ng/ml)	20 (6.9)	5 (10.9)	15 (6.2)	
Gravidity				
No pregnancies	7 (2.4)	1 (2.2)	6 (2.5)	0.269
1 pregnancy	58 (20.1)	9 (19.6)	49 (20.3)	
2 pregnancies	132 (45.8)	16 (34.8)	116 (47.9)	
3 or more pregnancies	91 (31.6)	20 (43.5)	71 (29.3)	
Oral contraceptive pill use (ever)				
Yes	220 (76.4)	39 (84.8)	181 (74.8)	0.203
No	68 (23.6)	7 (15.2)	61 (25.2)

**Table 2:** Geometric mean of heavy metals in blood by characteristics of study population

	Cadmium (μg/L)	*P*	Lead (μg/dL)	*P*	Mercury (μg/L)	*P*
	GM ± GSD		GM ± GSD		GM ± GSD	
All	0.98 ± 1.45		1.36 ± 1.41		1.61 ± 1.81	
Age						
<40 years old	0.94 ± 1.41	<0.001	1.33 ± 1.42	0.058	1.55 ± 1.80	0.046
≥40 years old	1.14 ± 1.50		1.46 ± 1.36		1.80 ± 1.82	
Education						
≤High school	1.00 ± 1.49	0.451	1.40 ± 1.40	0.267	1.67 ± 1.79	0.442
≥University	0.97 ± 1.43		1.34 ± 1.42		1.58 ± 1.82	
Income/monthly						
<4,000,000	1.00 ± 1.46	0.824	1.39 ± 1.41	0.166	1.54 ± 1.88	0.143
≥4,000,000	0.97 ± 1.43		1.33 ± 1.40		1.70 ± 1.72	
Body mass index						
Non-obesity (<25 kg/m^2^)	0.98 ± 1.42	0.479	1.36 ± 1.43	0.873	1.55 ± 1.81	0.010
Obesity (≥25 kg/m^2^)	0.99 ± 1.59		1.38 ± 1.30		2.01 ± 1.74	
Hemoglobin						
Anemia (<12 g/dL)	0.96 ± 1.42	0.002	1.35 ± 1.42	0.491	1.64 ± 1.79	0.118
Non-anemia (≥12 g/dL)	1.22 ± 1.62		1.42 ± 1.28		1.34 ± 1.93	
Serum cotinine						
No exposure (<10 ng/ml)	0.97 ± 1.45	0.024	1.36 ± 1.42	0.252	1.63 ± 1.82	0.123
Passive/Active/Heavy smoking (10 ≥ ng/ml)	1.12 ± 1.41		1.30 ± 1.35		1.36 ± 1.65	
Disease status						
Women without fibroids	0.97 ± 1.43	0.169	1.35 ± 1.41	0.334	1.59 ± 1.82	0.317
Women with fibroids	1.07 ± 1.52		1.43 ± 1.43		1.73 ± 1.76	
Fibroid size of women with fibroids						
Smaller than median size (<1.82cm^3^)	1.01 ± 1.51	0.839	1.36 ± 1.53	0.946	1.89 ± 1.66	0.433
Larger than median size (≥1.82cm^3^)	1.07 ± 1.55		1.45 ± 1.38		1.64 ± 1.81

**Table 3:** Associations between heavy metal levels and presence of uterine fibroids

	Unadjusted			Adjusted^a^		
	OR	95% CI	*P*	OR	95% CI	*P*
Cadmium (μg/L)	2.04	0.86-4.84	0.107	1.03	0.39-2.69	0.958
Mercury (μg/dL)	1.71	0.66-4.46	0.273	1.63	0.59-4.54	0.350
Lead (μg/L)	1.29	0.76-2.20	0.352	1.39	0.75-2.56	0.299

^a^Odds ratios (OR) adjusted for age, BMI, gravidity, oral contraceptive pill administration history, regularity of menstrual cycle, hemoglobin level, and serum cotinine levels

**Table 4:** Associations between heavy metal levels as continuous variable and volume of uterine fibroids

	The largest volume (cm^3^)					
	Unadjusted			Adjusted^a^		
	β	95% CI	*P*	β	95% CI	*P*
Cadmium (μg/L)	1.35	−0.17-2.86	0.080	2.22	0.06-4.37	0.044
Lead (μg/dL)	0.36	−1.42-2.14	0.687	0.12	−2.26-2.51	0.917
Mercury (μg/L)	−0.45	−1.65-0.75	0.450	−0.77	−2.30-0.75	0.309

^a^β coefficients adjusted for age, BMI, gravidity, oral contraceptive pill administration history, regularity of menstrual cycle, hemoglobin level, and serum cotinine levels

**Table 5:** Associations between heavy metal levels as categorical variable and volume of uterine fibroids

	Quartile	The largest volume (cm^3^)					
		Unadjusted			Adjusted^a^		
		β	95% CI	*P*	β	95% CI	*P*
Cadmium (μg/L)	1 (<0.9)	Ref.			Ref.		
	2 (0.9-1.0)	−0.91	−2.74-0.91	0.317	−0.47	−3.16-2.22	0.722
	3 (1.0-1.2)	−0.77	−2.60-1.05	0.396	−0.48	−3.12-2.16	0.713
	4 (1.2-3.1)	0.51	−1.32-2.34	0.575	1.19	−1.52-3.90	0.376
Lead (μg/dL)	1 (<1.1)	Ref.			Ref.		
	2 (1.1-1.3)	−0.39	−2.21-1.43	0.667	−0.42	−2.69-1.85	0.707
	3 (1.3-1.8)	0.85	−0.98-2.67	0.353	0.85	−1.67-3.37	0.496
	4 (1.8-3.2)	−0.77	−2.59-1.05	0.395	−1.23	−3.74-1.29	0.326
Mercury (μg/L)	1 (<1.2)	Ref.			Ref.		
	2 (1.2-1.7)	−0.54	−2.44-1.36	0.567	−0.23	−2.80-2.34	0.856
	3 (1.7-2.7)	−0.59	−2.48-1.31	0.535	−1.03	−3.58-1.52	0.415
	4 (2.7-4.7)	−0.40	−2.3-1.49	0.668	−0.65	−3.15-1.85	0.597

^a^β coefficients adjusted for age, BMI, gravidity, oral contraceptive pill administration history, regularity of menstrual cycle, hemoglobin level, and serum cotinine levels

## Discussion

We here demonstrated a significant relationship between blood cadmium concentrations and uterine fibroid volume by statistically adjusting for variables such as age, age at menarche, use of birth control pills prior to diagnosis, gravidity, regularity of menstrual cycle, BMI, hemoglobin level, and cotinine levels. To our knowledge, our findings are the first attempt to report the association of blood cadmium concentrations with uterine fibroid volume; our results show close agreement with the hypothesis that cadmium increases the volume of uterine fibroids.

These findings are similar to the findings of previous in vivo studies. Cadmium exposure is known to increase uterine wet weight and to proliferate human uterine leiomyoma cells based on a number of in vivo studies [24, 25]. Although the mechanisms are not clear, estrogen receptor (ER) stimulation and non-genomic activation of mitogen-activated protein kinase (MAPK) are considered possible biological mechanisms of cadmium’s actions [25, 26]. A number of studies have found more ERs in fibroids than in the homogeneous myometrium [27], and another study suggested that metalloestrogens activate the ER-α [11]. However, more recently there are several efforts to demonstrate that Cd-induced growths in human uterine leiomyoma and smooth muscle cells are not mediated by classical ER mechanism of receptor binding or estrogen response element-mediated gene activation [28], but by non-genomic pathways [27]. Non-genomic pathway involves differential activation of growth factor receptors and subsequent MAPK/extracellular signal-regulated kinase 1/2 phosphorylation [27]. Therefore, more studies are needed to demonstrate the exact mechanisms of estrogenic effects in cadmium exposure.

Unlike the results for blood cadmium, the blood lead and mercury levels were not significantly associated with fibroid volume in our study, which can be explained by a ranking of estrogenicity based on an in vitro study [12] that measured thee estrogenicity of major heavy metals by estrogen-receptor-dependent transcriptional expression assay. The results show the following estrogenicity ranking: cadmium chloride > lead acetate > lead nitrate > mercuric chloride [12]. Prior human studies regarding breast cancer and heavy metals also show this estrogenicity ranking [10]. Breast cancer is known to depend on estrogen, as uterine fibroids do. Studies of breast cancer and metalloestrogens have found major concerns with cadmium [10], and many epidemiological studies demonstrate the association between cadmium and breast cancer [29–31].

The increased volume of uterine fibroids is clinically important because most fibroids are asymptomatic [32], but larger fibroids can cause “bulk” symptoms including bowel and bladder dysfunction and abdominal pain [33]; consequently, large uterine fibroids need to be treated by surgery or medication. Because of this importance of uterine fibroid volume, our finding is medically meaningful for identifying strategies to prevent adverse effects of uterine fibroids.

In our study, although there was no clear statistical significance between the existence of uterine fibroids and heavy metals, the women with uterine fibroids showed higher geometric mean heavy metal concentrations and greater odds of having these three heavy metals in their blood than did the women without fibroids. Previously, two epidemiologic studies demonstrated the relationship between heavy metal blood concentrations and uterine fibroids [17, 18]. Research based on the National Health and Nutrition Examination Survey (NHANES) presented that uterine fibroids were not significantly associated with blood levels of heavy metals [17], whereas research based on an operative cohort of the Endometriosis: Natural History, Diagnosis, and Outcomes (ENDO) study showed the effects of blood cadmium and lead on uterine fibroids [18]. In terms of exposure, our study participants had higher blood concentrations of heavy metals than did participants in either of the previously reported two studies; our study participants showed, respectively, mean blood concentrations of 0.98 μg/L, 1.36 μg/dL, and 1.61 μg/L of cadmium, lead, and mercury, whereas in the NHANES, the participants showed means of 0.42 μg/L, 1.13 μg/dL, and 1.00 μg/L of cadmium, lead, and mercury, respectively [17]. In the ENDO study, the research participants who had uterine fibroids showed blood concentration means of 0.37 μg/L, 0.83 μg/dL, and 0.93 μg/L, and those without fibroids showed 0.30 μg/L, 0.61 μg/dL, and 0.55 μg/L of cadmium, lead, and mercury blood concentrations, respectively [18]. Regarding the study outcomes, the NHANES [17] is based on a self-reported questionnaire and thus determining whether the patients had uterine fibroids had limited sensitivity. The prevalence of uterine fibroids was 8% in the NHANES [17], much lower than the prevalence that had been previously reported from utilizing ultrasonography for the detection [2]. In contrast, ENDO study [18] showed uterine fibroid prevalence of 21% with the postoperative diagnosis. Although postoperative diagnosis clearly showed fibroids, small submucosal and intramural fibroids may have escaped detection. To diagnose the presence of fibroids, our study used both the questionnaire and pelvic ultrasonography, and prevalence was lower than expected (16.3%). Additionally, ENDO study [18] had more participants (*n* = 495) than our study (*n* = 282).

Our study does have a number of limitations; first, the study design was cross-sectional, and thus there was no temporality. Second, our participants consisted of healthy volunteers, and nearly all of them had given birth; thus, our study results could have been biased by this characteristic of the subjects. Third, our study population was smaller than that in previous studies, which could have reduced the statistical power in our study.

Although this study did have its limitations, it also has strengths. Our advanced diagnostic method with the questionnaire and the pelvic sonograms allowed us to confirm the history of myomectomy and asymptomatic uterine fibroids, respectively. This outstanding measurement method showed clearly reliable diagnosis results that were unattainable with the NHANES [17] and ENDO study [18]. This approach also enabled us to obtain the data on uterine fibroid volume, which allowed us to analyze the relationship between blood heavy metal concentrations and fibroid volumes.

## Conclusions

In summary, we attempted to determine the influence of heavy metals on the prevalence and volume of uterine fibroids. As demonstrated in our statistical results, which derived from a questionnaire and pelvic ultrasonography results, the women who had uterine fibroid showed higher geometric mean blood heavy metal concentrations as well as greater odds of having these three metals in their blood than the women who did not have fibroids. In addition, we also examined the relationship between uterine fibroid volume and the heavy metal concentrations. The blood cadmium concentration was significantly associated with fibroid volume, although the blood levels of lead and mercury were not.

Symptomatic uterine fibroids seriously reduce the quality of life in premenopausal women, and their volume has the most important influence on the symptoms. Therefore, well-designed studies considering the limitations of our study are needed for demonstrating the clear associations between heavy metal exposure and uterine fibroids in premenopausal women.

## Abbreviations

BMI: Body mass index
ENDO: Endometriosis: Natural History, Diagnosis and Outcomes
ER: Estrogen receptor
LOQ: Limit of quantification
MAPK: Mitogen-activated protein kinase
NHANES: National Health and Nutrition Examination Survey
